# Mixed colloids and acute kidney injury: a case of selection bias?

**DOI:** 10.1186/s13054-015-0797-9

**Published:** 2015-04-30

**Authors:** AB Johan Groeneveld, Roberta J Navickis, Mahlon M Wilkes

**Affiliations:** Department of Intensive Care, Erasmus Medical Center, 230 Gravendijkwal, 3015 CE Rotterdam, The Netherlands; Hygeia Associates, 17988 Brewer Road, Grass Valley, CA 95949 USA

In a previous issue of *Critical Care*, Frenette and colleagues [1] described a retrospective study of 984 cardiac surgery patients receiving mixed colloids, including hydroxyethyl starch (HES) 130/0.4 in 82% or HES 200/0.5 in 43%. In 25% or more of the patients, both HES solutions were used. A small minority (16%) received 5% or 25% albumin. An association was observed between albumin exposure and acute kidney injury (AKI).

Much essential information is not provided, including what colloids were administered, why, and when. How many patients received 5% albumin? 25% albumin? How often was albumin co-administered with HES 130/0.4? With HES 200/0.5? With both? In what co-administered doses? How much of each colloid was used for extracorporeal circuit priming? For volume expansion? How much albumin was used to correct hypoalbuminemia? What was the temporal relationship between albumin infusion and AKI development?

No serum albumin levels are reported. Yet preoperative hypoalbuminemia is a potent independent risk factor for renal failure in cardiac surgery patients (adjusted odds ratio 2.0, 95% confidence interval 1.3 to 3.2) [2]. A reported 15% of patients come to cardiac surgery with severe hypoalbuminemia (less than 25 g/L serum albumin), and the majority of those (58%) undergo elective procedures [2].

Albumin is typically reserved for a small minority of the highest-risk patients [3,4]. That practice may explain this study. The significantly lower HES 200/0.5 doses co-administered with albumin in the propensity-matched population suggest that albumin was substituted to mitigate the increased AKI risk of HES 200/0.5 previously reported by this same team of investigators [5]. With obvious potential selection bias and many unanswered questions, this study needs to be interpreted with caution.

## Abbreviations

AKI: Acute kidney injury
HES: Hydroxyethyl starch
