# Supplementary material for: Mixed colloids and acute kidney injury: a case of selection bias?
Source: Crit Care. 2015 Apr 30;19(1):207. doi: 10.1186/s13054-015-0797-9 (PMC4415346; doi:10.1186/s13054-015-0797-9)
Supplement: Related texts

# Reply: Authors’ response

**Authors:** Josée Bouchard, Anne Julie Frenette, Stéphan Troyanov, David R Williamson

We agree with Groeneveld and colleagues that there are several unanswered questions regarding the use of albumin. Albumin administration has been associated with different outcomes depending on the type of population studied [6-9]. In the recent Albumin Italian Outcome Sepsis (ALBIOS) trial, administration of 20% albumin did not improve prognosis by correcting hypoalbuminemia, improving hemodynamic status, or restricting the total amount of fluid administered [9]. In addition, albumin is expensive and may carry an infectious risk.

In our study, we acknowledged that we could not rule out residual confounding despite the use of a propensity score analysis and mentioned that we did not have data on serum albumin levels [1]. However, we described the timing and type of colloids administered and stated that the vast majority of the surgeries were elective. We used a propensity score analysis, which included the percentage and dose of synthetic colloids administered, to address any indication bias. However, our study does not provide a definitive answer on the administration of albumin in cardiac surgery patients. As we concluded, we believe that the safety of albumin-containing fluids on kidney function should be assessed in future cardiac surgery trials. The administration of a costly product currently associated with neutral or potentially negative outcomes in this population needs to be better delineated.
